# Indications and Complications of Tracheostomy in Children

**DOI:** 10.1590/S1808-86942010000300010

**Published:** 2015-10-20

**Authors:** Caroline Harumi Itamoto, Bruno Thieme Lima, Juliana Sato, Reginaldo Raimundo Fujita

**Affiliations:** 1 MD. Resident Physician – Department of Otolaryngology and Head and Neck Surgery; 2 MD. ENT physician, Fellow of the Otolaryngology and Head and Neck Department; 3 MSc. Graduate Student – Department of Otolaryngology and Head and Neck Surgery; 4 PhD. Adjunct Professor and Administrative Technician – Otolaryngology and Head and Neck Surgery Course

**Keywords:** postoperative complications, pediatrics, tracheostomy

## Abstract

Indications for tracheostomy have changed and its complications are more common in children.

**Aim:** To evaluate the indications and complications of tracheostomies performed in children.

**Materials and Methods:** A retrospective study with review of medical records of patients aged from 1 day to 16 years who underwent tracheostomy at a university hospital during the period of August 2000 to July 2008. We assessed data on age, gender, indications and intra and postoperative complications.

**Results:** Fifty-eight children under 16 years of age underwent tracheotomy during the study period. The mean age was 3.7 years. Airway obstruction was the main indication for surgery (n = 40; 69%). The incidence of complications in the postoperative period was 19% (11 patients), the majority happening during the late postoperative period. A further complication observed was cannula clogging. There were no complications related to the procedure during the surgery.

**Conclusion:** The main indications for tracheostomy in children were airway obstruction and prolonged OTI. The most frequent postoperative complications were cannula obstruction and decannulation. The indications and complications observed were similar to those reported in the literature by other services.

## INTRODUCTION

Tracheostomy is one of the oldest medical procedures known^1^; it has been routinely used since the middle of the 19th century, when Armand Trousseau improvised the technique in order to treat diphtheria patients with dyspnea^2^.

The indication of tracheostomy in children has changed substantially in the last two decades. The upper airway obstruction of infectious origin was the main reason for which children were submitted to tracheostomy, most of the time in an emergency basis^3^. Today, the main indications are: prolonged orotracheal intubation (OTI), upper airway obstruction caused by craniofacial malformations (such as the Robin's sequence, Treacher-Collins syndrome, Beckwith-Wiedemann syndrome, Nager syndrome and the CHARGE association), laryngotracheal stenosis and hypoventilation associated with neurologic disorders, such as brain palsy4. Since the survival of children with these congenital and neurological disorders is on the rise, a greater number of tracheostomies are being done in such population.

In pediatric patients this procedure is more challenging and it is associated to a higher degree of morbidity and mortality when compared to the adult population5. The younger the child is submitted to the procedure, the greater is the risk of complication^3^,^5^.

The goal of the present study is to analyze the indications and complications associated with tracheostomies carried out in children in a University Hospital between July of the year 2000 and August of 2008.

## MATERIALS AND METHODS

This was a historical, cross-sectional cohort from the medical charts of children submitted to tracheostomy in a University Hospital, between August, 2000 and July of 2008.

A list of all the patients submitted to tracheostomy during this period was provided by the Anesthesiology Department of the Hospital. For each and every surgical procedure carried out in the hospital there is an anesthesia report, in which the anesthesiologist records data regarding the name, gender, age and hospital number of the patient, surgery date and indication, whether it was urgent or not, the type of anesthesia and the surgeon in charge. After the surgery, the anesthesia report is sent to the Anesthesiology Program Department, where the data is then recorded in a Microsoft Excel file.

The hospital where the study was carried out is a tertiary care center, with a pediatric ICU, high risk maternity, genetic services, pediatric craniofacial and otolaryngology service, being a reference center for other facilities in the treatment of high complexity disorders.

From the list provided by the Department of Anesthesiology, we selected children from 1 day of life to 16 years of age. After reviewing the charts obtained from the medical files, we surveyed the indications and intra and postoperative complications of the tracheostomies carried out during such period, being assessed globally and according to the age range: neonates (up to 28 days of life), infants (up to 2 years of life), pre-school aged (2-6 years), school-aged children (7-9 years), adolescents (10 years old or more).

In total, 73 children were submitted to tracheostomy; nonetheless, we had insufficient data from 58 patients.

This study was approved by the Ethics Committee of the University Hospital, protocol # 0171/2006.

## RESULTS

### Demographics

**Graph 1:**
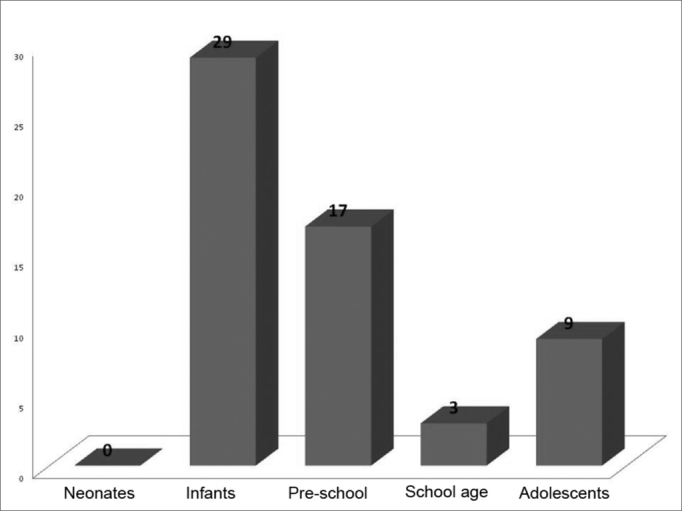
Number of children operated by age range.

Of the 58 children studied, 25 (43.1%) were girls and 33 (56.9%) were boys. The youngest was 1 month old and the oldest was 16 years old (mean age of 3.7 years). None of the children were operated during the neonatal period, 29(50%) were infants, 17(29.3) pre-school aged, 3(5.2%) school aged and 9(15.5%) were adolescents. These results are depicted on Graph 1.

**Graph 2:**
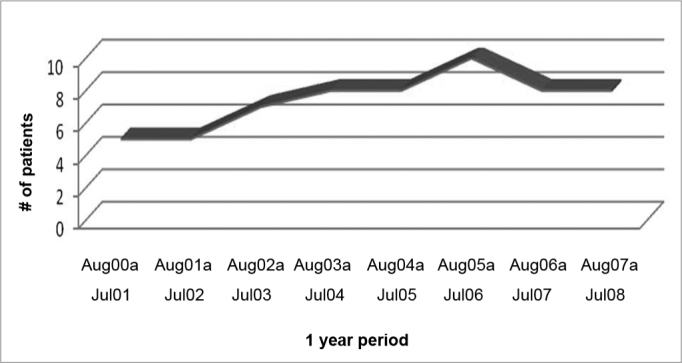
Number of patients per period

The number of tracheostomies done per year varied and showed a growing trend, peaking between August of 2005 and July of 2006, which can be seen on Graph 2.

### Indications

Indications for tracheostomies were: upper airway obstruction (n=40; 69%), prolonged OTI (n=14; 24%), severe Sleep Obstructive Apnea Syndrome (SOAS) (n=2; 3.4%), pulmonary clearance (n=1; 1.8%) and airway protection in the post-op of total thyroidectomy with bilateral lymph node dissection (n=1; 1.8%). Such indications are shown on Table 1.

**Table 1:** Indications for tracheostomy.

Indication	Patients (%)
Airway obstruction	40 (69%)
Prolonged OTI	14 (24%)
Severe SOAS	2 (3,4%)
Pulmonary clearance	1 (1,8%)
Protective tracheostomy	1 (1,8%)
TOTAL	58 (100%)

Among the causes for airway obstruction, most of them were caused by laryngotracheal stenosis (32; 80%). Of these, 28 (87.5%) had had prolonged OTI, one patient (3.1%) had congenital stenosis and three (9.4%) had history of multiple laryngeal microsurgeries in order to resect papillomas. The patients with acquired laryngotracheal stenosis had had OTI for 25 days (mean value).

The diagnoses of the patients submitted to tracheostomy for airway obstruction are depicted on Table 2.

**Table 2:** Diagnosis of the patients submitted to tracheostomies because of airway obstruction.

Diagnosis	Patients (%)
Laryngotracheal stenosis	32 (80%)
Recurrent laryngeal papillomatosis	2 (5%)
Severe laryngomalacia	2 (5%)
Pharyngeal tumor	2 (5%)
Mucopolysaccharidosis	1 (2,5%)
Bilateral vocal fold paralysis	1 (2,5%)
TOTAL	40 (100%)

Patients with severe laryngomalacia had genetic syndrome and cerebral palsy associated. The patient with bilateral vocal fold paralysis also had Arnold-Chiari malformation. The pharyngeal tumors of those patients submitted to tracheostomy were lymphangioma and fibromatosis.

Among the patients submitted to tracheostomy for prolonged OTI, the intubation time varied between 7 and 120 days (mean of 57 days).

One patient with cerebral palsy required tracheostomy for pulmonary clearance purposes.

In two cases, the tracheostomy was indicated because of severe SOAS, one patient had mucopolysaccharidosis and pulmonary hypertension and the other patient had achondroplasia.

In one patient, the tracheostomy was carried out as a means of protection, because the patient had been submitted to total thyroidectomy and bilateral cervical lymph node dissection.

In three cases the tracheostomy was carried out in an emergency basis, because of ventilation and intubation difficulties during anesthesia induction – one of these patients had mucopolysaccharidosis and two had laryngeal papillomas.

Among the infants, the indications for tracheostomy were: airway obstruction (n=18; 62%) and prolonged OTI (n=11; 38%). The main indications in the pre-school aged children were airway obstruction (n=13; 76.5%) and SOAS (n=2; 11.8%). Among school-aged children, indications were: airway obstruction (n=2; 66.7%) and prolonged OTI (n=1; 33.3%) and among adolescents: airway obstruction (n=7; 77.8%) and prolonged OTI (n=2; 22.2%), as depicted on Table 3.

**Table 3:** Indications for tracheostomy in each age group.

Age group	Indications	n (%)
Neonates	–	–
	Airway obstruction	
	- Laryngotracheal stenosis	16(55.2%)
Infants	- Bilateral vocal fold paralysis	1(3.4%)
	- Pharyngeal tumor	
	TOTAL	18(62%)
	Prolonged OTI	11(38%)
	Airway obstruction	
	- Laryngotracheal stenosis	11(64.7%)
Pre-school age	- Laryngomalacia	1(5.9%)
	- Laryngeal papillomatosis	1(5.9%)
	TOTAL	13(76.5%)
	SOAS	2(11.7%)
	Pulmonary clearance	1(5.9%)
	Protective tracheostomy	1(5.9%)
	Airway obstruction	
School age	- Laryngotracheal stenosis	1(33.35%)
	- Laryngeal papillomatosis	1(33.35%)
	TOTAL	2(66.7%)
	Prolonged OTI	1(33.3%)
	Airway obstruction	
	- Laryngotracheal stenosis	4(44.5%)
Adolescents	- Mucopolysaccharidosis	1(11.1%)
	- Laryngomalacia	1(11.1%)
	- Pharyngeal tumor	1(11.1%)
	TOTAL	7(77.8%)
	Prolonged OTI	2(22.2%)

Among infants, the indications for tracheostomy were: airway obstruction (n=18; 62%) and prolonged OTI (n=11; 38%). The main indications among pre-school aged children were airway obstruction (n=13; 76.5%) and SOAS (n=2; 11.8%). Among school-aged children, indications were: airway obstruction (n=2; 66.7%) and prolonged OTI (n=1; 33.3%) and among adolescents: airway obstruction (n=7; 77.8%) and prolonged OTI (n=2; 22.2%). Such results are depicted on Table 3.

### Complications

Complications from the procedure were seen in 19% (11 in 58) of the patients. Of these, 1 (9.1%) happened in the immediate post-op (IPO), in other words, within the first 24 hours after surgery; 3 (27.3%) in the recent post-op (recent PO), in other words, within the first week after surgery; and 7 (63.6%) happened in the late post-op (LPO). There were no intraoperative complications. The complications seen were: cannula obstruction (n=5), accidental cannula loss (n=3), death (n=2), aspiration bronchopneumonia (n=2), tracheomalacia (n=1), pneumothorax (n=1) and false course of the cannula (n=1). Some patients had more than one complication. Such complications can be seen on Table 4.

**Table 4:** Complications per period

Period	Complication	n(%)
Intraoperative	–	–
Immediate PO	Aspiration bronchopneumonia + septic shock+ death	1(9.1%)
	Accidental cannula loss + false course intubation + pneumothorax	1(9.1%)
Recent PO	Cannula obstruction	1(9.1%)
	TOTAL	3(27.3%)
	Cannula obstruction	4(36.3%)
	Accidental cannula loss	1(9.1%)
Late PO	Aspiration bronchopneumonia	1(9.1%)
	Tracheomalacia	1(9.1%)
	TOTAL	7(63.6%)

**Graph 3:**
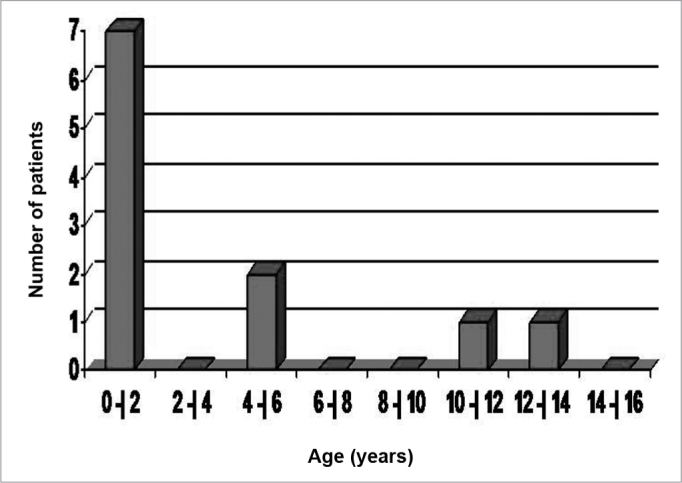
Number of patients per age range.

Cannula obstructions happened because of papilloma in two cases, secretion in two cases and granuloma in one case. All successfully treated.

One of the accidental cannula loss cases happened during the IPO and peaked with hypoventilation and death. Another patient had accidental cannula loss in the recent post-op, followed by false course, pneumothorax and cardio-respiratory arrest – which were reverted. The third case of accidental decannulation happened in the LPO and did not have greater repercussions.

One patient had aspiration bronchopneumonia in the recent post-op and developed septic shock and death; the other case of aspiration pneumonia happened in the LPO and was clinically treated, with a favorable outcome.

The mean age of the patients who had complications was 3.9 years, most of them were infants (63.6%), as per depicted on Graph 3.

## DISCUSSION

The main indication for tracheostomy in the pediatric population from the hospital where the study was carried out was airway obstruction, responsible for 69% of the procedures, while prolonged OTI was the cause of 24% of the surgeries. Despite the variability in indications seen in the present study, we have noticed changes in the indications for tracheostomies in children along the years^6^,^7^.

During the 70's, the main indication was of infectious origin, including epiglottitis and laryngotracheobronchitis. Starting during the 80's, acute epiglottitis and laryngotracheobronchitis were no longer common indications for tracheostomy because of the introduction of OTI and the vaccine against type B Haemophilus influenzae, responsible for acute epiglottitis^7^,^8^. In our study, there was no procedure done because of acute airway infections. Most of the procedures were carried out in patients with chronic disorders.

Laryngomalacia, laryngotracheal stenosis and recurrent laryngeal papillomatosis are causes of respiratory obstruction in children, being seen among the children submitted to tracheostomy in our study.

Laryngomalacia treatment is based on observation and follow up in most cases, since symptoms tend to spontaneously disappear before the second year of life. On the other hand, in some cases, the disease has a bad outcome (severe laryngomalacia) and surgical intervention becomes necessary. Numerous surgical procedures are described for the treatment of this disease, among them we have supraglottoplasty, section or partial resection of the aryepiglottic folds and epiglottopexy. Nonetheless, some patients with severe laryngomalacia do not improve with surgical treatment, and then it is necessary to use non-invasive ventilation associated with tracheostomy. Children with neuropathies usually have unfavorable outcome and worse response to conventional surgical treatment, as seen among the patients in our study and who required tracheostomy.

In cases of laryngotracheal stenosis, it is indicated to observe and follow the patient up when the obstruction does not cause respiratory distress to the child, nor interferes in his/her daily activities, feeding, sleep, growth and development. Nonetheless, in most of the cases, tracheostomy is needed because of the respiratory distress, in order to secure the airway in the post-op or when the age of the patient prevents surgical reconstruction – when it is necessary to wait for the child to grow in order to carry out the surgery.

The treatment of the recurrent laryngeal papillomatosis is based on papilloma exeresis in order to keep the airway patent. However, in some cases, the disease is highly recurrent, resulting in airway obstruction, and then tracheostomy is necessary.

In a study carried out with 122 children from the Starship Pediatric Hospital in New Zealand, between 1987 and 2003^9^, they also reported that the airway obstruction was the main reason for doing a tracheostomy (70%), followed by prolonged OTI (30%). In this study, the incidence of complications was 51%, concentrated in the late postop period (43%), especially because of peri-tracheostoma granuloma formation.

In a review study involving 208 children from the King's Daughters Pediatric Hospital in the US, between 1988 and 1998^4^, the airway obstruction was the indication in only 19% of the cases. Nonetheless, the incidence of 32% if craniofacial abnormalities were included in the airway obstruction group. In a study carried out in Lyon (France), involving 46 children from the Edouard Herriot University Hospital, between 1996 and 2001, the airway obstruction and OTI were indications in 43 and 57% of the cases, respectively^10^.

In our study, 51.7% of the procedures were carried out in infants, being compatible with other papers, such as the one from Carron et al.^5^, with 55%, and Donnelly et al.^7^, with 48%. Because of progresses in pediatric care we have seen recently, especially in intensive care centers, and the introduction of increasingly more efficient antibiotic agents, there has been an increase in the survival of neonates and premature babies, including those with congenital anomalies and, consequently, the number of tracheostomies in this age range.

We have noticed that, throughout the years, the incidence of tracheostomies has increased, shown by the study from Mahadevan et al.^9^ and by a review carried out in the Our Lady Hospital (Ireland), between 1971 and 1990^7^. Such increase can be explained by the larger number of children who need tracheostomies because of congenital anomalies or for needing chronic ventilation, since the survival of these children has improved in recent years, according to Datasus.

The general incidence of complications in our study was 19%, which proved favorable in relation to other similar studies, which incidence varied between 31 and 44%^3^,^4^,^7^,^11^. In these, most of the complications also happened during the LPO, varying between 23 and 35%, having cannula obstruction and granuloma formation as the main causes.

In children, the mortality associated with tracheostomy in the literature varied between 0.5 and 3%, and has accidental decannulation and cannula obstruction as the main causes^12^. In the population we studied, one death (1.7%) was directly related to the surgery (accidental cannula loss) and the other was a consequence of aspiration bronchopneumonia which evolved with septic shock.

With improvements in the surgical technique along the years, the development of care programs for tracheostomized patients and improvements in the post-op follow up, a drop in tracheostomy-related complications is expected. The integration of a multidisciplinary team, including nursing and physical therapy, as we have in our settings, has helped much to enhance the quality of life and also the survival of these tracheostomized children. In our pediatric population, tracheostomies have proven to be a relatively safe procedure, which main function is to maintain the airway properly patent for ventilation.

## CONCLUSION

In the last eight years, the main indications for tracheostomies in children were airway obstruction and prolonged OTI. The most frequent post-op complications were cannula obstruction and accidental decannulation. Most of the patients who developed complications were 2 years old or younger.
